# Genetic and pharmacological inhibition of vanin-1 activity in animal models of type 2 diabetes

**DOI:** 10.1038/srep21906

**Published:** 2016-03-02

**Authors:** Janna A. van Diepen, Patrick A. Jansen, Dov B. Ballak, Anneke Hijmans, Floris P.J.T. Rutjes, Cees J. Tack, Mihai G. Netea, Joost Schalkwijk, Rinke Stienstra

**Affiliations:** 1 Department of Internal Medicine, Radboud University Nijmegen Medical Centre, 6525 GA Nijmegen, The Netherlands; 2 Radboud Institute for Molecular Life Sciences, Radboud University Nijmegen Medical Centre, Nijmegen, 6525 GA Nijmegen, The Netherlands; 3 Institute for Molecules and Materials, Radboud University Nijmegen, 6525 GA Nijmegen, The Netherlands; 4 Nutrition, Metabolism and Genomics Group, Division of Human Nutrition, Wageningen University, 6703 HA, Wageningen, the Netherlands

## Abstract

Vanins are enzymes that convert pantetheine to pantothenic acid (vitamin B5). Insights into the function of vanins have evolved lately, indicating vanin-1 to play a role in inflammation, oxidative stress and cell migration. Moreover, vanin-1 has recently gained attention as a novel modulator of hepatic glucose and lipid metabolism. In the present study, we investigated the role of vanin-1 in the development of hepatic steatosis and insulin resistance in animal models of obesity and diabetes. In addition, we evaluated the potency of RR6, a novel pharmacological vanin-1 inhibitor, as an anti-diabetic drug. Increased vanin activity was observed in plasma and liver of high fat diet (HFD)-induced obese mice, as well as ZDF-diabetic rats. Ablation of vanin-1 (*Vnn1*^−/−^ mice) mildly improved glucose tolerance and insulin sensitivity in HFD-fed mice, but had no effects on body weight, hepatic steatosis or circulating lipid levels. Oral administration of RR6 for 8 days completely inhibited plasma vanin activity, but did not affect hepatic glucose production, insulin sensitivity or hepatic steatosis in ZDF-diabetes rats. In conclusion, absence of vanin-1 activity improves insulin sensitivity in HFD-fed animals, yet short-term inhibition of vanin activity may have limited value as an anti-diabetic strategy.

Disturbances of hepatic glucose and lipid metabolism play an important role in the development of insulin resistance and type 2 diabetes. Vanin-1 has recently gained interest as a novel modulator of hepatic glucose- and lipid metabolism, and has been suggested as a potential target to treat metabolic diseases^1^,^2^. Vanin-1 is an enzyme with pantetheinase activity, which catalyzes the hydrolysis of pantetheine into pantothenic acid (vitamin B5) and cysteamine^3^,^4^. Vanin-1 is member of a larger vanin family, consisting of three human (VNN1, VNN2 and VNN3) and two mouse (Vnn1 and Vnn3) orthologous genes^5^. Although vanins are poorly characterized at the functional level, insights into the function of vanin-1 have evolved lately, largely due to generation of the Vnn1-deficient mouse^6^. So far, vanins have been indicated to play a role in inflammation, oxidative stress and cell migration^7^,^8^,^9^, processes that are thought to be mediated via vanin-dependent cysteamine production^10^.

In addition, several recent studies have elucidated a role for vanin-1 in the regulation of key metabolic pathways. Expression of vanin-1 is high in liver and strongly induced by fasting, as one of the most prominently regulated genes by PPARα^1^,^11^. Moreover, after prolonged fasting, inhibition of vanin activity aggravates hepatic steatosis^1^. Interestingly, increased vanin-1 expression has recently also been observed in murine steatotic livers during obesity^12^,^13^. Whether inhibition of vanin activity is causally involved in the development of steatosis during obesity remains unknown.

In addition to hepatic lipid metabolism, vanin-1 was recently shown to modulate glucose metabolism by directly enhancing hepatic glucose output^2^. These observations point towards vanin-1 inhibition as a potential novel pharmacological target to treat metabolic diseases with enhanced glucose production, such as diabetes.

In the current study we set out to study the effect of vanin-1 deficiency on the development of obesity-induced hepatic steatosis and diabetes, as well as the potency of RR6, a novel pantetheinase (vanin) inhibitor, as an anti-diabetic drug using experimental animal models.

## Results

### The presence of obesity and insulin resistance promotes vanin activity in animal models

**Figure 1:**
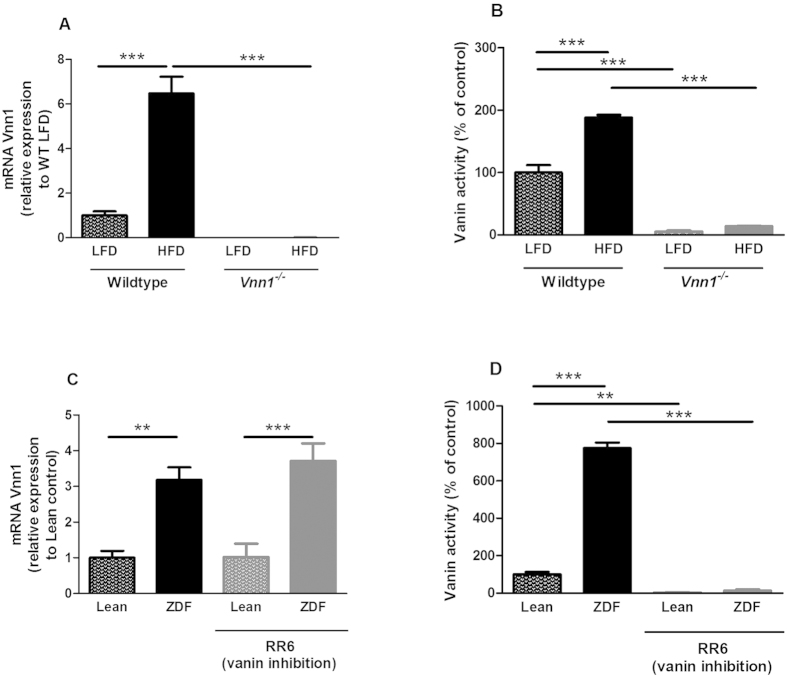
Obesity and insulin resistance increases vanin activity. Vnn-1^−/−^ and wild type (WT) mice were fed low fat diet (LFD) or high fat diet (HFD) for 16 weeks. Depicted are: (**A**) relative hepatic mRNA levels of Vnn1 and (**B**) plasma vanin activity. Data are mean ± SD from n = 7–9 animals per group. Lean and ZDF rats were treated with the vanin inhibitor RR6. (**C**) Relative hepatic mRNA levels of Vnn1 and (**D**) plasma vanin activity were determined. Data are mean ± SD from n = 5 animals per group. *p < 0.05, ** P < 0.01, *** P < 0.001.

The levels of vanin activity were evaluated in HFD-induced obese (DIO) mice as well as ZDF-diabetes rats. Both hepatic vanin-1 mRNA (Fig. 1A) and plasma vanin activity (Fig. 1B) were increased in DIO mice as compared to the LFD-fed lean controls. Similarly, ZDF-diabetes rats displayed augmented hepatic vanin-1 mRNA expression (Fig. 1C) and increased plasma vanin activity (Fig. 1D) as compared to lean controls. In contrast to vanin-1, expression of vanin-3 was only mildly upregulated in obese, insulin-resistant mice and rats (Supplementary Fig. S1A,B).

### Metabolic effects of diet-induced obesity in vanin-1 knockout mice

**Figure 2:**
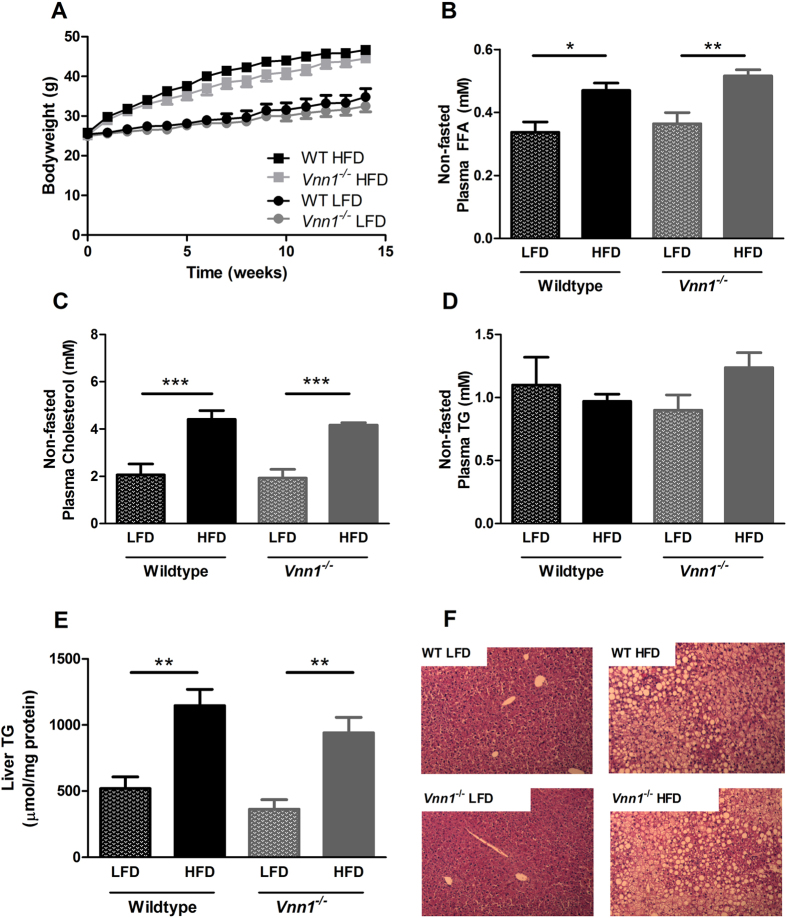
Plasma lipids and hepatic steatosis in diet-induced obese vanin-1 knockout mice. Vnn-1^−/−^ and wild type (WT) mice were fed low fat diet (LFD) or high fat diet (HFD) feeding for 16 weeks. (**A**) Bodyweight development and non-fasted (**B**) plasma free fatty acids, (**C**) plasma cholesterol, (**D**) plasma triglycerides (TG), (**E**) hepatic triglyceride [TG] content and (**D**) H&E staining of liver sections; magnification 200×. Data are mean ± SD from n = 7–9 animals per group. *p < 0.05, ** P < 0.01.

Plasma vanin activity was strongly reduced in Vnn-1^−/−^ mice (−93%; Fig. 1B). To study the causal relation between vanin-1 and obesity-related metabolic disturbances, Vnn-1^−/−^ and WT mice were fed 16 weeks high-fat diet (45% kcal, HFD) or a control low-fat diet (10% kcal, LFD). HFD-feeding induced a similar bodyweight in both Vnn-1^−/−^ and WT mice (Fig. 2A), and food intake was not different between both genotypes (Supplementary Fig. S2A). In addition, plasma cholesterol and free fatty acid (FFA) levels were increased upon HFD-feeding, but not affected by vanin-1 deficiency (Fig. 2B,C). Plasma TG did not differ between groups (Fig. 2D). Recent studies, as well as our data, show a strong increase in vanin-1 expression in murine steatotic livers (Fig. 1)^12^,^13^, prompting to hypothesize a causal role for vanin-1 in the progression of steatosis^13^. However, we show that hepatic steatosis as reflected by hepatic TG levels (Fig. 2E) and HE staining (Fig. 2F) was not affected by vanin-1 deficiency in LFD- or HFD-fed mice.

### The absence of vanin-1 mildly improves insulin sensitivity in obese, insulin resistant mice

**Figure 3:**
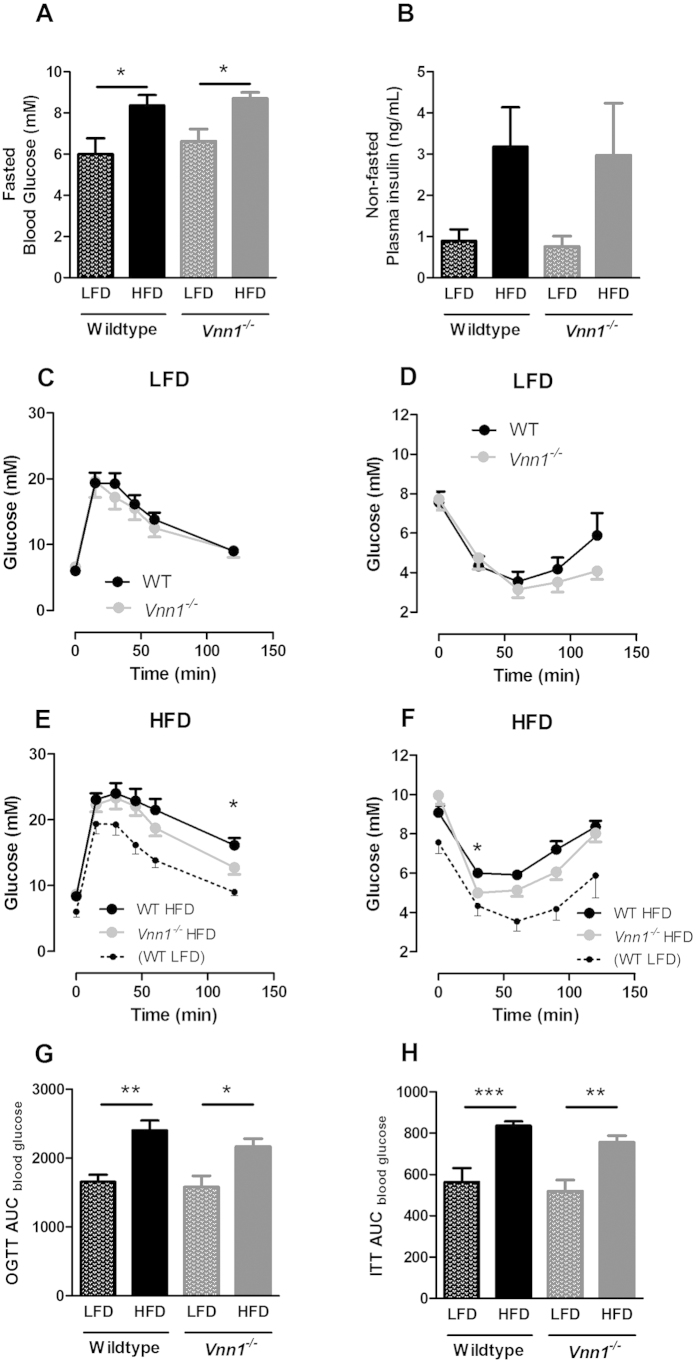
The absence of vanin-1 mildly improves insulin sensitivity in obese, insulin resistant mice. Vnn-1^−/−^ and wild type (WT) mice were fed low fat diet (LFD) or high fat diet (HFD) feeding for 16 weeks. (**A**) 9h Fasted blood glucose and (**B**) non-fasted plasma insulin levels after 16 weeks of LFD or HFD. Blood glucose levels of LFD-fed mice during (**C**) oral glucose tolerance test (OGTT) and (**D**) insulin tolerance test (ITT), as well as HFD-fed mice during (**E**) OGTT and (F) ITT. Area under the curve (AUC) during OGTT (**G**) and ITT (**H**). Data are mean ± SD from n = 7–9 animals per group. *p < 0.05.

Subsequently, we evaluated the role of vanin-1 in obesity-induced disturbances in glucose metabolism. Therefore, LFD- and HFD-fed Vnn-1^−/−^ and WT mice were subjected to an oral glucose tolerance test (OGTT) and insulin tolerance test (ITT). Absence of vanin-1 neither affected plasma glucose nor insulin levels in mice fed a LFD or HFD (Fig. 3A,B). Upon LFD-feeding, no difference was observed between Vnn-1^−/−^ mice and WT mice regarding glucose tolerance (Fig. 3C) or insulin sensitivity (Fig. 3D). Upon HFD-feeding, however, Vnn-1^−/−^ mice had a slightly faster glucose clearance as compared to WT littermates, as reflected by lower glucose levels 2h after an oral glucose load (Fig. 3E). In addition, insulin injection induced a faster drop in glucose levels in Vnn-1^−/−^ versus WT mice fed a HFD (Fig. 3F). Despite the slightly faster insulin-induced clearance of glucose, total area under the curves for OGTT and ITT were not different between HFD-fed *Vnn1*^−/−^ and WT mice (Fig. 3G,H). These data suggest that a reduction in vanin activity mildly improved insulin sensitivity in an obese, insulin-resistant mouse model.

Absence of vanin-1 has been shown to reduce inflammation and oxidative stress during infection of injury^14^,^15^. Since these pathways are known to contribute to insulin resistance, expression of markers of inflammation and oxidative stress in liver and adipose tissue were determined (Supplementary Fig. S3). High-fat feeding similarly increased expression of *F4/80, Clec7a* and *Tnfα* in adipose tissue of Vnn1^−/−^ and WT mice (Supplementary Fig. S3A–C), accompanied by a similar reduction in Glut4 expression (Supplementary Fig. S3D), suggestive of comparable levels of inflammation and insulin resistance in adipose tissue in both genotypes. Similarly in liver, Vanin-1 deficiency did not affect expression of genes involved in inflammation (F4/80, Clec7a, IL-1b; Supplementary Fig. S3E–G) or oxidative stress (*Gss, Gpx1, Gclm*; Supplementary Fig. S3H–J). Thus, the mild improvement in insulin sensitivity in HFD-fed *Vnn1*^−/−^ is not associated with apparent changes in inflammation or oxidative stress in liver or adipose tissue.

### The effect of pharmacological inhibition of vanin activity in ZDF-diabetes rats

We subsequently evaluated whether RR6, a recently developed competitive inhibitor of vanin activity^16^, would improve insulin sensitivity in an animal model for diabetes.

RR6 supplementation in drinking water is a potent inhibitor of vanin activity in rats^16^, almost completely inhibiting plasma vanin activity (Fig. 1D and Supplemental Fig. S4). In contrast in mice, no complete inhibition of vanin activity is obtained by RR6 administration (max 75%; Supplemental Fig. S4) due to a very fast clearance (half life approx. 80 min). Since no complete vanin inhibition could be achieved in mice, we used the ZDF-diabetes rats as an animal model for diabetes to evaluate the effects of RR6 on hepatic steatosis and insulin sensitivity.

**Figure 4:**
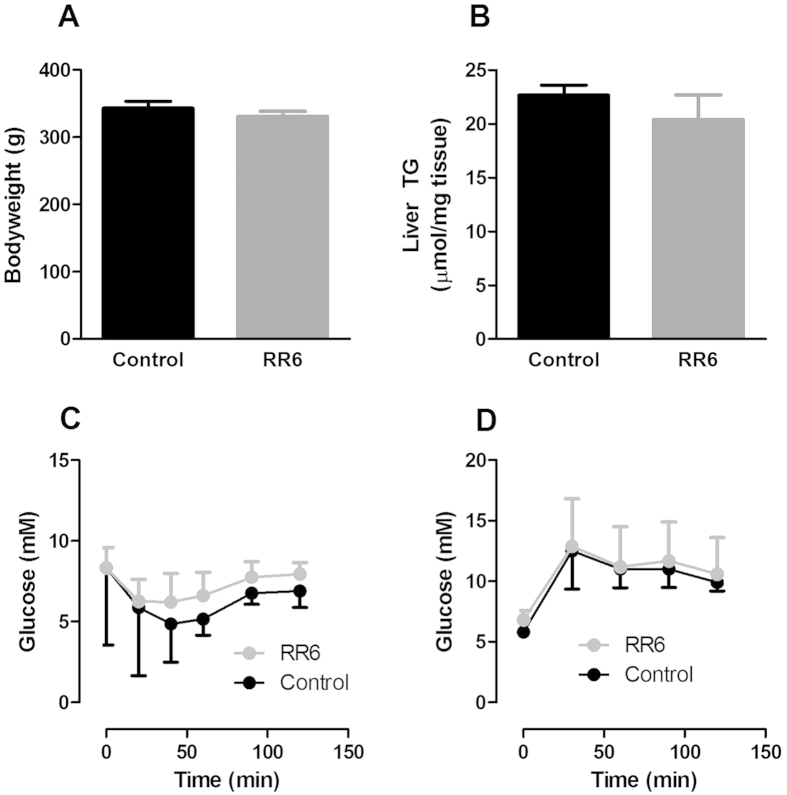
The effect of pharmacological inhibition of vanin activity in ZDF-diabetes rats. ZDF rats were administered with the vanin inhibitor RR6 in drinking water for 8 days. Depicted are (**A**) bodyweight, (**B**) hepatic triglyceride [TG] content and blood glucose levels during (**C**) ITT and (**D**) pyruvate tolerance test (PTT). Data are mean ± SEM from n = 5 animals per group.

Treatment of ZDF-diabetes rats with RR6 for 8 days led to an almost complete inhibition of plasma vanin activity (−98%; Fig. 1D). Similar to mice, inhibition of vanin activity did not affect bodyweight (Fig. 4A), food intake (Supplementary Fig. S2B) or hepatic steatosis as measured by TG levels (Fig. 4B), supported by similar expression of hepatic PPARγ (Supplementary Fig. S5A,D). In addition, vanin inhibition did not affect plasma levels of TG, total cholesterol or FFA levels (Supplementary Fig. S6A–C). In contrast to the results obtained in mice, we found no effect of vanin-inhibition on insulin sensitivity in ZDF-diabetes rats (Fig. 4C). It has recently been shown that inhibition of vanin-1 with siRNA reduces expression of genes involved in hepatic gluconeogenesis and reduces fasting plasma glucose levels^2^. Therefore, to determine whether vanin inhibition by RR6 affects hepatic gluconeogenesis in obese, diabetic rats, we performed a pyruvate tolerance test, which allows for quantification of gluconeogensis. Inhibition of vanin activity did not affect fasting glucose levels, or hepatic glucose production during a pyruvate test (Fig. 4D). In accordance, vanin-1 deficiency in mice or inhibition of vanin activity in rats did not affect hepatic expression of the gluconeogenic genes *Pepck* or *G6pase* (Supplementary Fig. S5).

## Discussion

Vanin-1 is known as a pantetheinase, catalyzing the hydrolysis of pantetheine into pantothenic acid and cysteamine. In addition, vanin-1 recently emerged as one of the most prominent genes regulated by PPARα^1^,^11^,^17^ and is proposed to play a role in the regulation of key metabolic pathways^10^. Especially under fasting conditions, vanin activity has been reported to modulate lipid- and glucose metabolism and could be a new therapeutic target in metabolic disease^1^,^2^,^16^.

The first objective of the current study was to examine whether absence of vanin-1 or inhibition of vanin activity, would affect hepatic steatosis in obese animal models. After prolonged fasting, inhibition of vanin activity aggravates hepatic steatosis^1^. In addition, the highly increased vanin-1 expression in murine steatotic livers^12^,^13^, instigated the idea of a causal role for vanin-1 in the progression of steatosis during obesity. However, our experiments show that neither genetic absence (*Vnn1*^−/−^ mice) nor pharmacological inhibition of total vanin activity by RR6 affects hepatic steatosis in animal models of obesity. Thus vanin-1 expression appears to prevent excessive accumulation of TG in the liver during fasting^1^, but not in response to HFD-feeding. This suggests that the observed increase in vanin-1 expression in steatotic livers of obese mice, may rather be a marker of enhanced PPARα activation, without causal involvement of vanin-1 in the progression or prevention of steatosis during obesity. Our data show that the increased expression of Vnn1 in steatotic livers concurs with an increase in total plasma vanin activity. Therefore, plasma vanin activity could possibly serve as a valuable marker of hepatic PPARα activation and/or steatosis in obese subjects. In human subjects, vanin activity has shown to increase in response to both prolonged fasting as well as treatment with fibrates, known ligands for PPARα^1^. Whether vanin activity can serve as a marker for steatosis in obese subjects remains to be established.

The current experiments show that absence of vanin-1 did not affect hepatic expression of genes involved in inflammation or oxidative stress in HFD-fed mice. Importantly however, HFD-fed C57Bl/6 mouse and the Zucker Diabetic Fatty (ZDF) rat are models to evaluate development of hepatic steatosis, but not development of steatohepatitis or hepatic oxidative stress^18^,^19^. Therefore, the effect of vanin-1 on these pathological hepatic processes needs careful future evaluation in more appropriate experimental settings. The HFD-fed C57Bl/6 mouse is a suitable mouse model to evaluate development of adipose tissue inflammation during obesity. Indeed, HFD-feeding in the current study clearly induced adipose tissue inflammation. Vanin-1 has previously been shown to antagonize peroxisome proliferator-activated receptor gamma activity in epithelial cells^7^. A similar mechanism in adipocytes would hypothetically improve adipose tissue function and reduce inflammatory pathways. However, our data show that absence of vanin-1 did not affect adipose tissue inflammation or function.

The second objective of the current study was to examine whether absence of vanin-1 or inhibition of vanin activity, would improve insulin sensitivity in obese animal models. To this extend, glucose metabolism was investigated in HFD-fed *Vnn-1*^−*/*−^ mice as well as in ZDF rats treated with the novel vanin-inhibitor RR6. Although we observed a strong upregulation of hepatic *Vnn1* expression and plasma vanin activity in obese, insulin resistant mice and rats, our results suggest that vanin-1 only plays a minor role in the pathophysiology of insulin resistance. Whereas the complete absence of vanin-1 led to an increase in insulin sensitivity and glucose tolerance after a long term HFD-intervention in mice, short-term inhibition of vanin activity had no beneficial effect on insulin sensitivity or hepatic glucose production in diabetic rats. These results suggest that the contribution of vanin-1 to the development of insulin resistance is rather indirect. Another explanation for the differences observed in RR6-treated rats and Vnn1^−/−^ mice could possibly be that not vanin activity, but the presence of vanin-1 protein may be (mildly) involved in the development of insulin resistance. The recently revealed structure of vanin-1 by X-ray crystallography suggests that it could operate both as an enzyme and a signaling protein^20^. In our ZDF-rats model, vanin inhibition did not affect the expression of vanin-1 (Fig. 1C) or vanin-3 (Supplementary Fig. S1B), which could thus still function as signaling proteins.

Previous findings show that basal glucose levels and glucose tolerance do not change in *Vnn1*^−/−^ mice^21^, which is line with our observations in control LFD-fed *Vnn1*^−/−^ and WT mice. Our findings in HFD-fed mice are partly in contrast with Chen *et al*., who demonstrated increased hepatic glucose production and insulin resistance upon activation of vanin-1 using adenovirus vanin-1 (adVnn1), while inhibition of vanin-1 using siRNA against VNN1 reduced these processes in an obese mouse model^2^. We did not observe regulation of genes involved in gluconeogenesis, such as *Pepck* or *G6pase*, in *Vnn1*^−/−^ animals or after treatment with RR6. Possibly, differences in the dietary intervention approach or in the animal models that were used may explain the discrepancies between the two studies. Additionally, the effect of liver-specific and short-term (3 days) modulation of vanin-1 activity by siRNA done by Chen *et al*. versus total inhibition of vanin-1 activity in our study may have differential outcomes on systemic glycemic control. But most importantly, treatment of *db/db* mice with siVNN1 reduced their food intake by 50% 2, which will largely affect their bodyweight (which were not shown) and contribute to the observed reduction in blood glucose levels. Importantly, the improved glucose tolerance and insulin sensitivity induced by siVNN1 in their study was largely defined by a significant difference in basal glucose levels, while the change in blood glucose levels after insulin or glucose injection were similar^2^. In our hands, absence of vanin or inhibition of vanin activity did not affect food intake. Moreover, for therapeutic possibilities, the current results on pharmacological inhibition of vanin activity are likely most relevant.

We did not observe any effect of vanin-1 deficiency on plasma TG, cholesterol or FFA levels in mice upon HFD-feeding. Interestingly, pantethine, the stable disulfide of pantetheine and substrate for vanin activity, is known as a natural compound with hypolipidemic effects^22^, which may be mediated by increased cysteamine levels^23^. Importantly, the HFD-fed C57Bl/6 mouse model and ZDF rat models were used to evaluate the effect of vanin inhibition on hepatic steatosis and insulin resistance specifically. These animal models are characterized by only minor changes in plasma lipid levels upon HFD-feeding. Therefore, to specifically evaluate the effect of vanin(-1) inhibition on plasma lipids and lipoprotein metabolism, additional studies need to be performed in more suitable hyperlipidemic mouse models such as the ApoE3Leiden or LDLr deficient mouse model.

In conclusion, our findings indicate that absence of vanin-1 only mildly improves insulin sensitivity, whereas short-term inhibition of vanin-activity using RR6 has no beneficial effects in obese, insulin resistant animals.

## Methods

### Animals and RR6 administration

Mice and rats were housed under standard conditions and experiments were approved by the institutional ethical committee on animal experimentation of the Radboud University Medical Center (Nijmegen) and methods were carried out in accordance with the approved guidelines. Vanin-1 knockout mice were generated as described before^6^ and were backcrossed to a C57Bl/6J background. Male vanin-1^−/−^ (*Vnn1*^−*/*−^) mice and wild type (WT) littermates of 12–14 weeks old were given low-fat (LFD) or high-fat diet (HFD) diet for 16 weeks, containing 10% or 45% of energy derived from fat, respectively (D12450B or 12451; Research Diets, Inc). Male lean Wistar rats and Zucker Diabetic Fatty (ZDF) rats (Harlan) of 8 weeks old were treated with the pantetheinase (vanin) inhibitor RR6 (3 mg/mL; dissolved in drinking water^16^) or vehicle for 8 days. The pantetheine analogue RR6 acts as a selective and potent vanin inhibitor, which causes an almost complete inhibition of all vanin activity in rats^16^. Mice and rats were sacrificed at the end of the diet- or RR6 treatment respectively (non-fasted; between 9 am and 11 am) and blood and organs were collected for analysis. Pharmacodynamic analysis of RR6 in mice was performed and compared to RR6 pharmacodynamics in rats that have been described before^16^. Shortly, animals (n = 3) were given RR6 by a single oral administration (50 mg/kg; dissolved in 10% DMSO in PBS). Heparinized blood samples were drawn at several time points after the oral gavage and plasma was obtained by centrifugation for determination of plasma vanin activity as described below.

### Studies on glucose homeostasis

Oral glucose tolerance (OGTT), insulin tolerance (ITT) and pyruvate tolerance tests (PTT) were performed. Prior to the OGTT, animals were fasted overnight (9 hours; 11 pm–8 am) after which 2 g/kg glucose (D-glucose, Gibco, Invitrogen) was orally administered. Prior to the ITT and PTT, animals were fasted 4 hours (5 am–9 am) and insulin (0.75 U/kg) or pyruvate (1.5 gr/kg) was injected intraperitoneally. Blood glucose levels were determined with an Accu-chek glucosemeter (Roche Diagnostics, Almere, The Netherlands) at stated time points.

### Vanin activity assay

Vanin (pantetheinase) activity was determined in plasma as described before^16^,^24^. Shortly, a buffered pantothenate-7-amino-4-methylcoumarin solution (Pan-AMC; 10 μM final concentration) was evaporated to dryness in 96-well plates before plasma samples were added and incubated for 60 minutes. Fluorescence was measured using a luminescence spectrometer (LS55, Perkin Elmer).

### Plasma and hepatic lipid analysis

Non-fasted plasma levels of triglycerides (TG), total cholesterol (TC) (Liquicolor, Human GmbH, Wiesbaden, Germany) and free fatty acids (FFA; NEFA-C WAKO chemicals, GmbH, Neuss Germany) were determined enzymatically according to manufacturer’s instructions. Hepatic TG concentrations were measured in 10% liver homogenates using a commercial kit from Liquicolor (Human GmbH, Germany) and expressed per mg tissue. For histological examination, H&E staining of sections was done using standard protocols on 5 μm-thick liver sections.

### RNA isolation and qPCR analysis

Total RNA was isolated from liver and adipose tissue using TRIzol (Invitrogen, Carlsbad, CA), according to manufacturer’s instructions. RNA was reverse transcribed (iScript cDNA Synthesis Kit, Bio-Rad Laboratories) and qPCR was performed using Power SYBR green master mix (Applied Biosystems, Foster City, CA) using the Step-one Real-Time PCR system (Applied Biosystems). Values were corrected using the housekeeping gene 36B4. Primer sequences are listed in Supplemental Table 1.

### Statistical analysis

Data are means ± SD unless indicated otherwise. Statistical analyses were performed using Graphpad Prism 5.0. Differences between 4-groups were tested with ANOVA with post-hoc Bonferroni correction. Comparisons between 2-groups (RR6 vs control) were calculated using a Student *t*-test. P < 0.05 was considered statistically significant.

## Additional Information

**How to cite this article**: van Diepen, J. A. *et al*. Genetic and pharmacological inhibition of vanin-1 activity in animal models of type 2 diabetes. *Sci. Rep.* **6**, 21906; doi: 10.1038/srep21906 (2016).

## Supplementary Material

Supplementary Information
