# Supplementary material for: Genetic and pharmacological inhibition of vanin-1 activity in animal models of type 2 diabetes
Source: Sci Rep. 2016 Mar 2;6:21906. doi: 10.1038/srep21906 (PMC4773925; doi:10.1038/srep21906)
Supplement: Supplementary Information [file srep21906-s1.doc]

**Genetic and pharmacological inhibition of vanin-1 activity in animal models of type 2 diabetes**

**Janna A. van Diepen^1^,*, Patrick A. Jansen^2^, Dov B. Ballak^1^, Anneke Hijmans^1^, Floris P.J.T. Rutjes^3^, Cees J. Tack^1^, Mihai G. Netea^1^, Joost Schalkwijk^2^, Rinke Stienstra^1,4^**

^1^ Department of Internal Medicine, Radboud University Nijmegen Medical Centre, 6525 GA Nijmegen, The Netherlands

^2^ Radboud Institute for Molecular Life Sciences, Radboud University Nijmegen Medical Centre, Nijmegen, 6525 GA Nijmegen, The Netherlands

^3^ Institute for Molecules and Materials, Radboud University Nijmegen, 6525 GA Nijmegen, The Netherlands

^4^ Nutrition, Metabolism and Genomics Group, Division of Human Nutrition, Wageningen University, 6703 HA, Wageningen, the Netherlands

**Supplemental Figure S1:** Obesity and insulin resistance only mildy upregulates Vnn3 expression in mice and rats

**Supplemental Figure S2:** Food intake in diet-induced obese *Vnn-1*^-/-^ and wild-type mice and ZDF-rats treated with RR6

**Supplemental Figure S3:** Absence of vanin-1 does not affect expression of genes involved in inflammation and oxidative stress in adipose tissue and liver of diet-induced obese mice

**Supplemental Figure S4:** Pharmacodynamics of vanin inhibition by RR6 in mice and rats.

**Supplemental Figure S5:** Hepatic qPCR analysis in diet-induced obese *Vnn-1*^-/-^ and wild-type mice and ZDF-rats treated with RR6.

**Supplemental Figure S6:** Plasma lipid levels in ZDF-rats treated with RR6.

**Supplemental Table S1:** Primers used for quantitative real-time PCR analysis


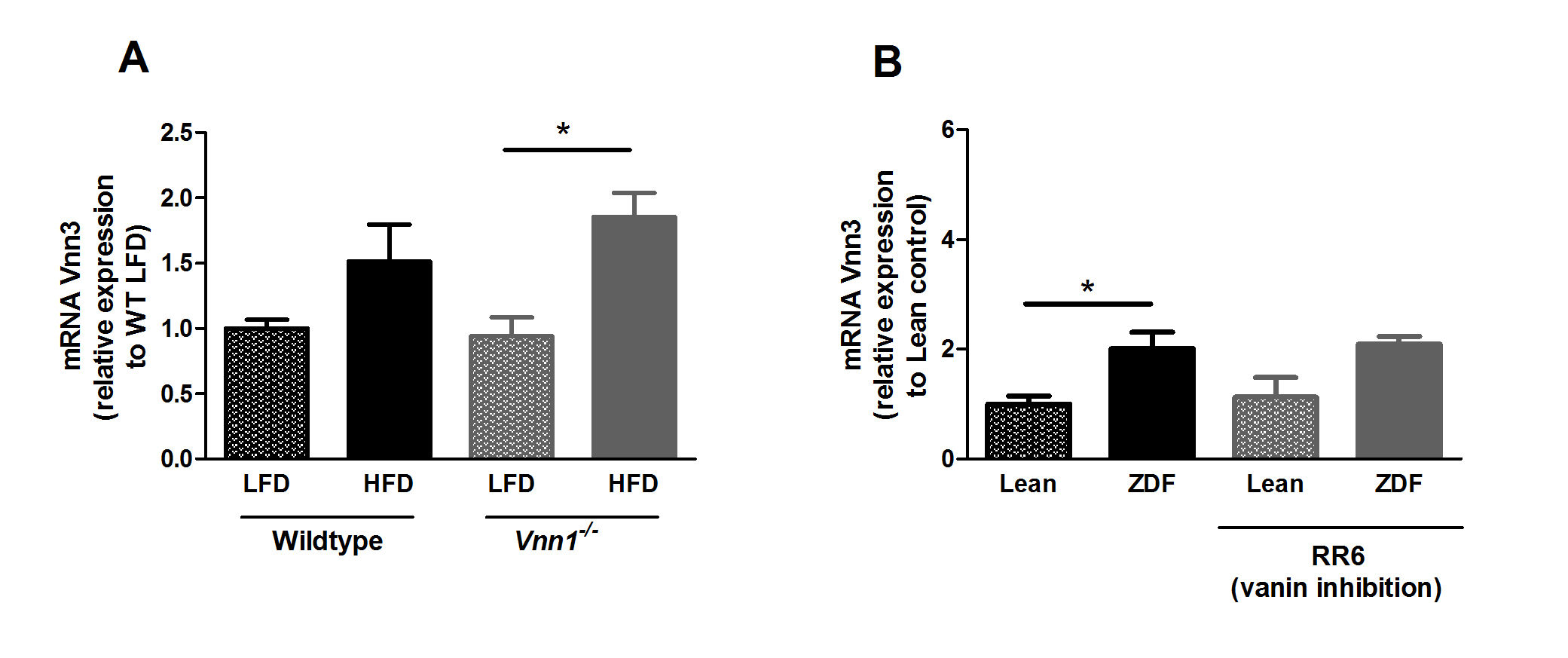


**Supplemental Figure S1: Obesity and insulin resistance only mildy upregulates Vnn3 expression in mice and rats**

Vnn-1^-/-^ and wild type (WT) mice were fed low fat diet (LFD) or high fat diet (HFD) feeding for 16 weeks. Depicted are (A) relative hepatic mRNA levels of Vnn3. Data are mean ± SEM from n=7-9 animals per group. Lean and ZDF rats were treated with the vanin inhibitor RR6. (B) Relative hepatic mRNA levels of Vnn3 were determined. Data are mean ± SEM from n=5 animals per group.


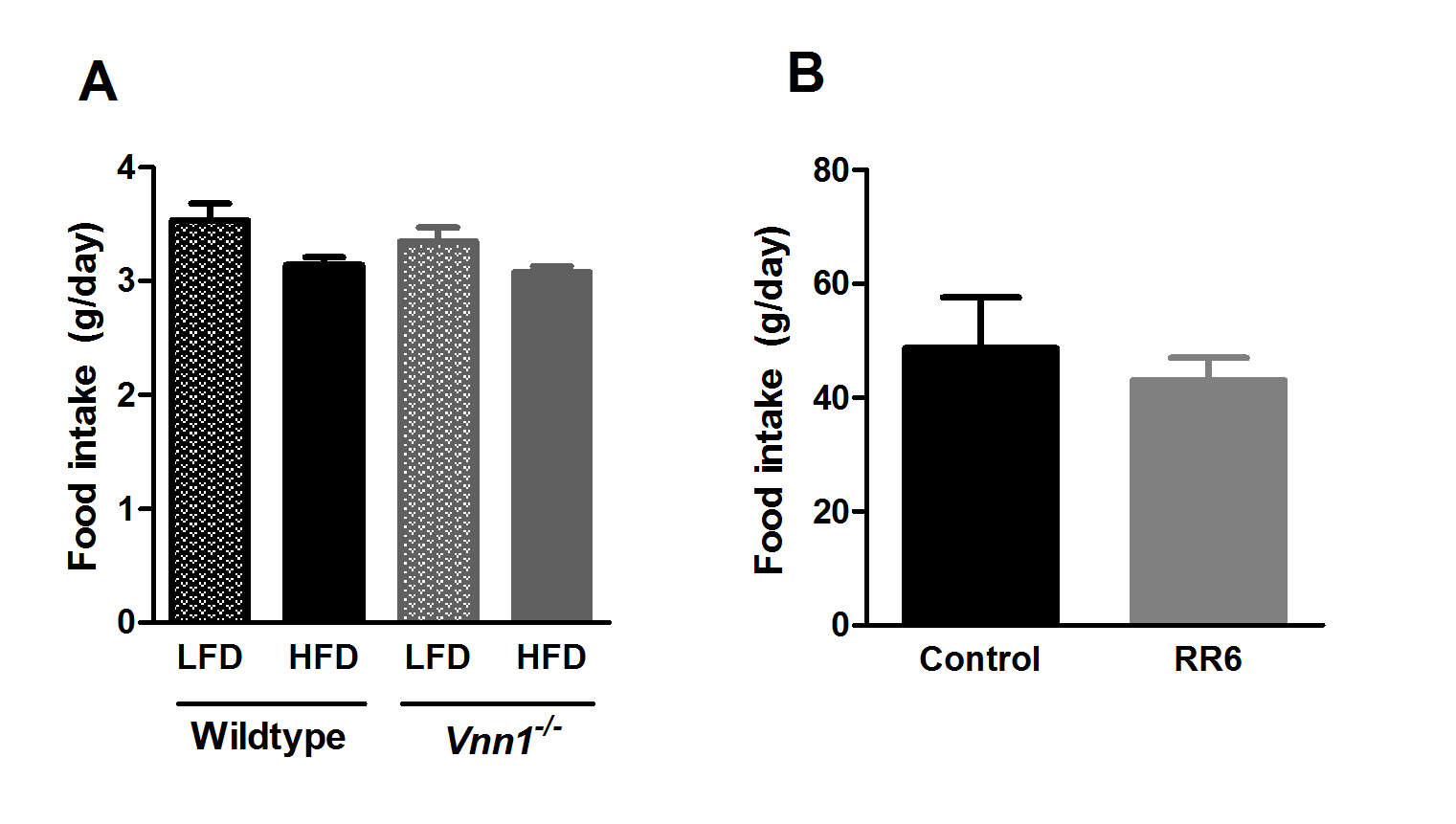


**Supplemental Figure S2: Food intake in diet-induced obese *Vnn-1*^-/-^ and wild-type mice and ZDF-rats treated with RR6**

Average food intake was recorded for (A) Vnn-1^-/-^ and wild type (WT) mice fed low fat diet (LFD) or high fat diet (HFD) feeding for 16 weeks and (B) lean and ZDF rats treated with the vanin inhibitor RR6. Data are mean ± SEM.


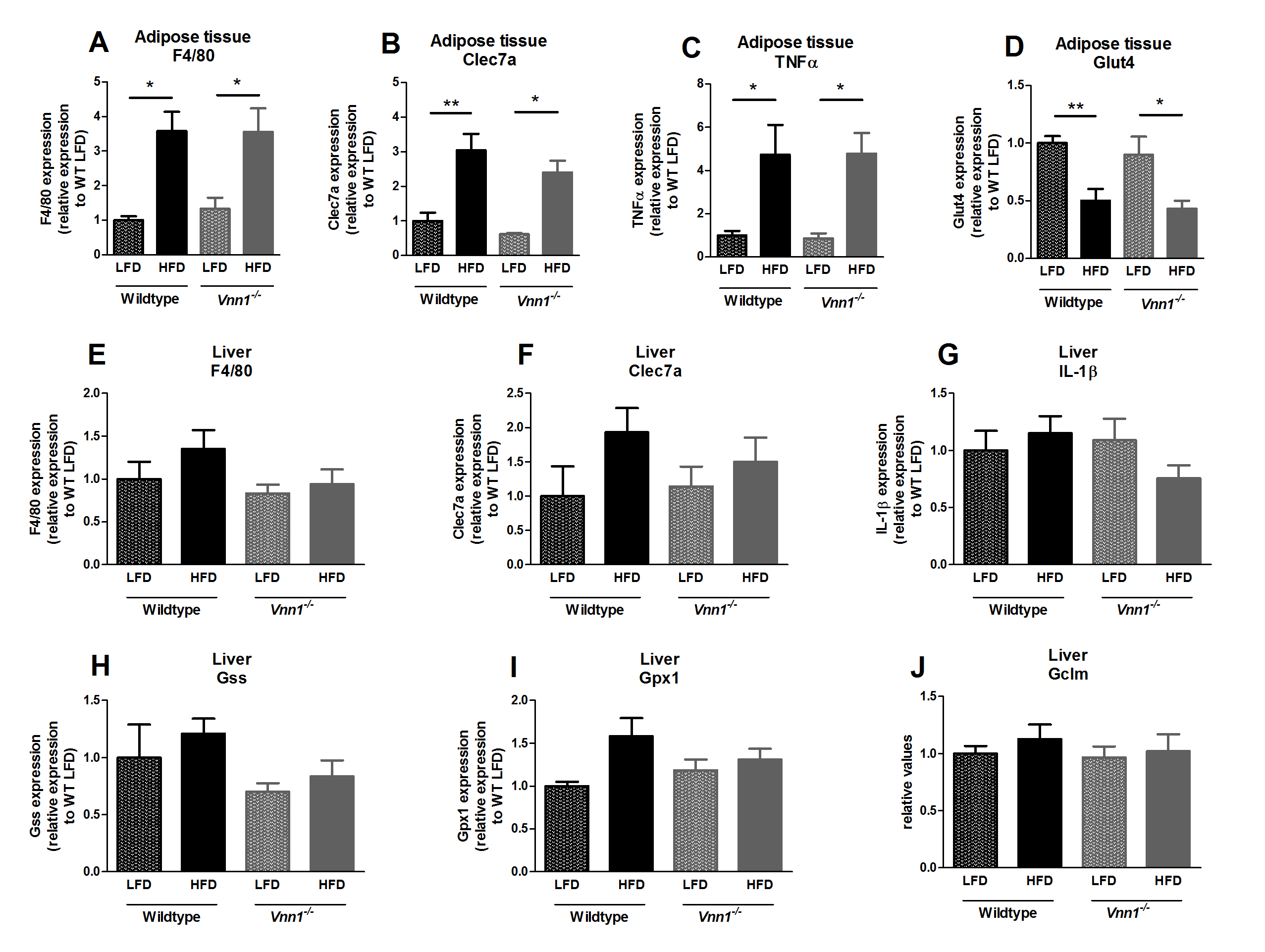


**Supplemental Figure S3:** **Absence of vanin-1 does not affect expression of genes involved in inflammation and oxidative stress in adipose tissue and liver of diet-induced obese mice.** Liver and adipose tissue were isolated from *Vnn1*^-/-^ and wild-type mice fed a low-fat diet (LFD) or high fat diet (HFD) for 16 weeks. Adipose tissue expression of (A) F4/80, (B) Clec7a, (C) TNFα and (D) Glut4 were determined and expressed as fold-change compared to the LFD fed wild-type mice. Hepatic expression of inflammatory markers (E) F4/80, (F) Clec7a, (G) IL-1β and oxidative stress markers (H) Gss, (I) Gpx1 (J) Gclm were determined and expressed as fold-change compared to the control treated rats. Data are mean ± SEM from n=5-9 animals per group. * P<0.05, ** P<0.01


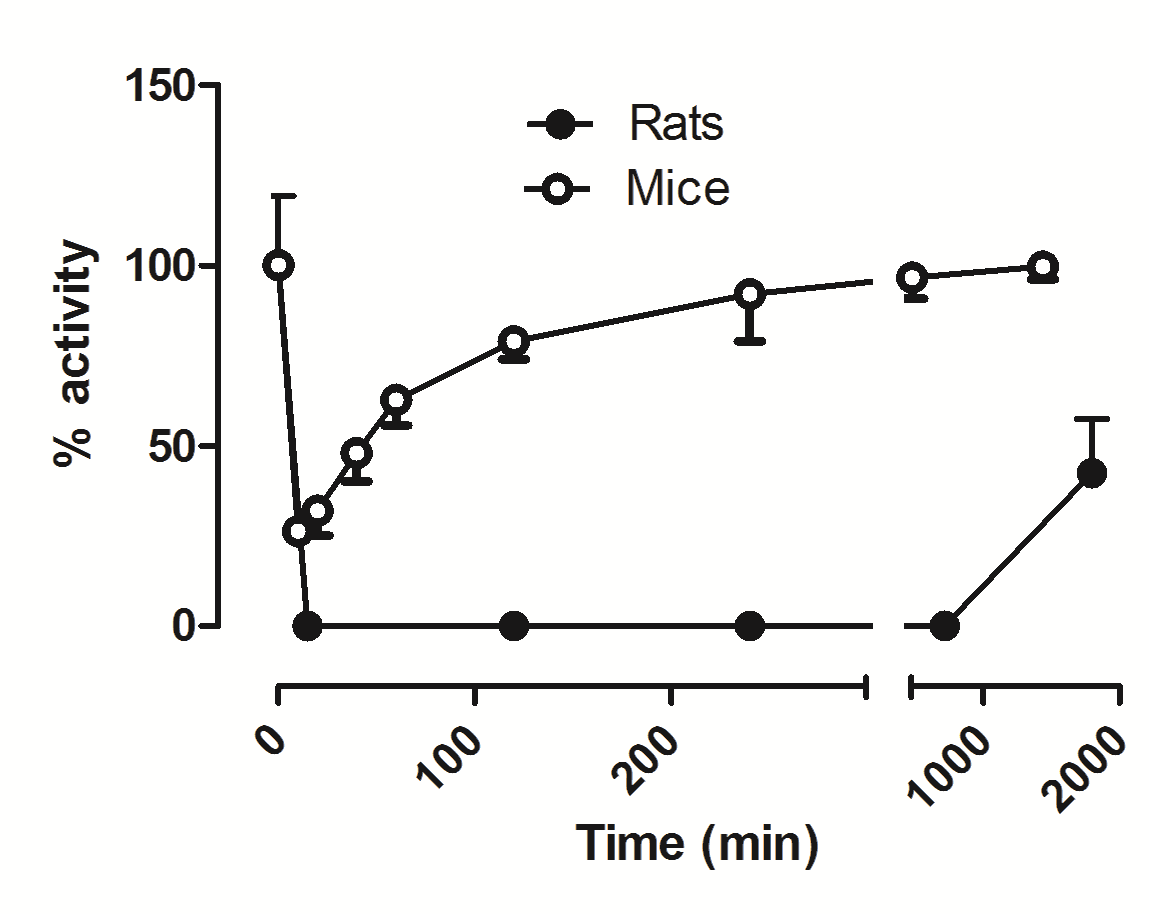


**Supplemental Figure S4:** **Pharmacodynamics of vanin inhibition by RR6 in mice and rats.** RR6 was orally given to wild-type mice and rats, blood samples were collected in time and vanin activity was measured. Oral administration of the vanin inhibitor RR6 results in a stronger and prolonged inhibition of plasma vanin activity in rats as compared to mice. The data on pharmacodynamics of RR6 in rats have been published before and are used for reference here (Jansen, ACS Chem Biol 2013). Data are mean ± SEM from n=3 animals per group.

*Reference:* Jansen, P. A. M. *et al.* Discovery of Small Molecule Vanin Inhibitors: New Tools To Study Metabolism and Disease. *Acs Chem Biol* 8, 530-534 (2013).


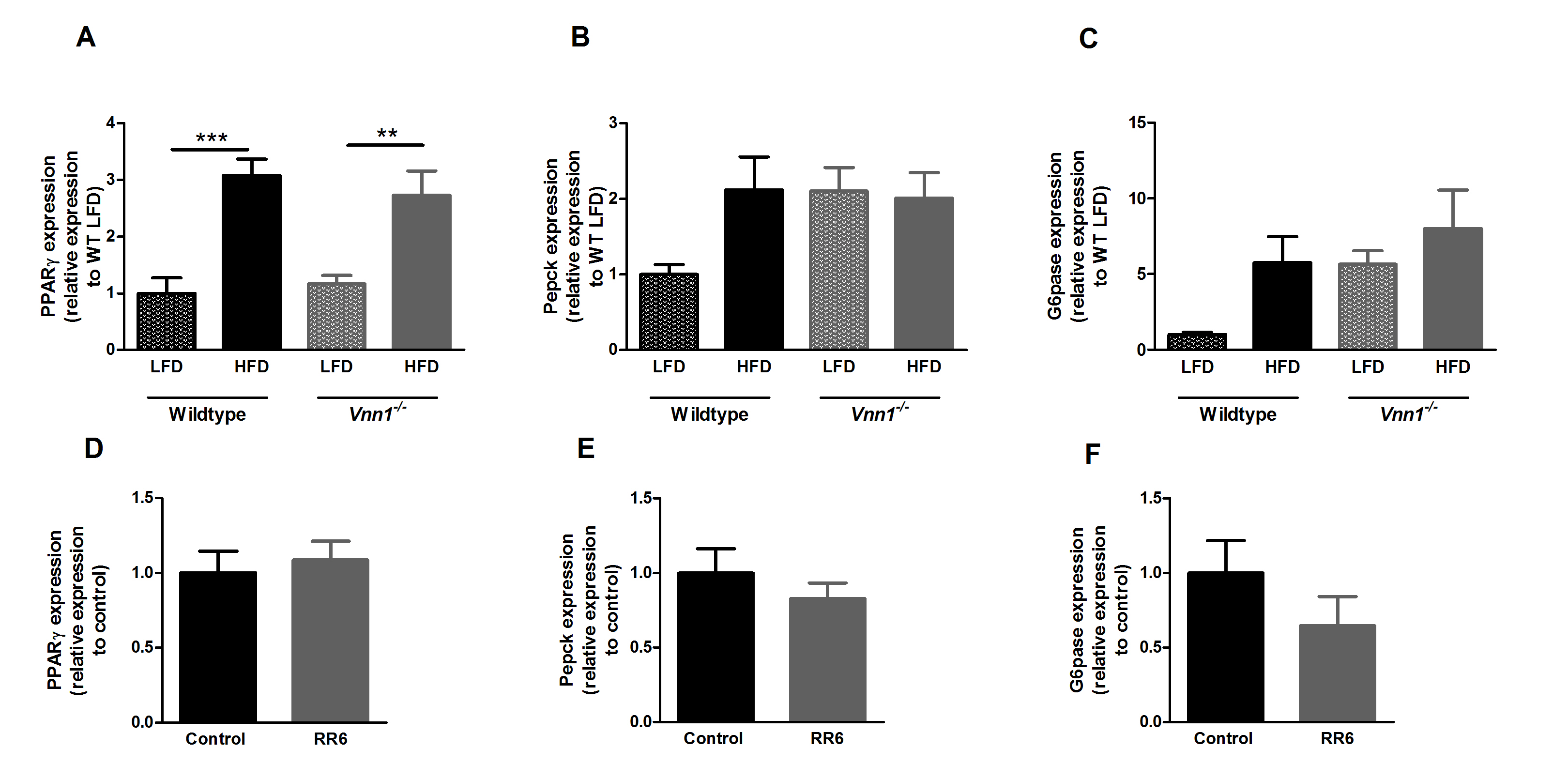


**Supplemental Figure S5:** **Hepatic qPCR analysis in diet-induced obese *Vnn-1*^-/-^ and wild-type mice and ZDF-rats treated with RR6.** Livers were isolated from *Vnn1*^-/-^ and wild-type mice fed a low-fat diet (LFD) or high fat diet (HFD) for 16 weeks. Expression of (A) Pparg (B) Pepck and (C) G6pase was determined and expressed as fold-change compared to the LFD fed wild-type mice. ZDF rats were administered with the vanin inhibitor RR6 in drinking water for 8 days and livers were isolated. Expression of (D) Pparg (E) Pepck and (F) G6pase was determined and expressed as fold-change compared to the control treated rats. Data are mean ± SEM from n=5-9 animals per group. ** P<0.01, *** P<0.001


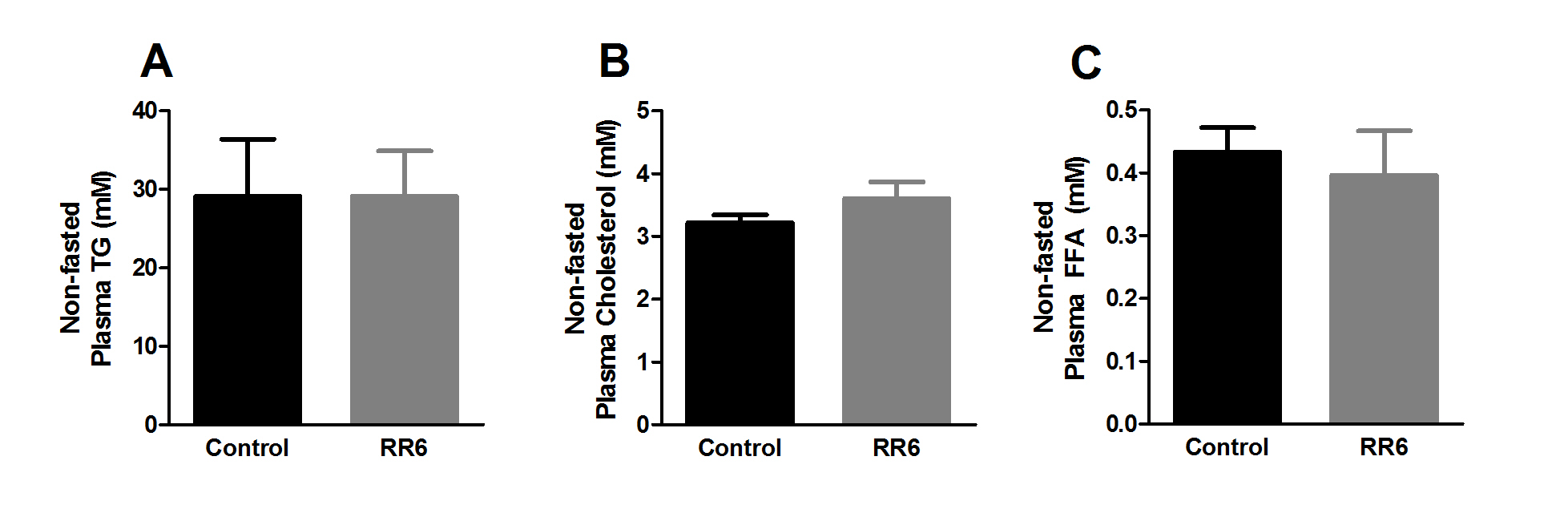


**Supplemental Figure S6:** **Plasma lipids levels in ZDF-rats treated with RR6**

ZDF rats were administered with the vanin inhibitor RR6 in drinking water for 8 days and non-fasted plasma was isolated. (A) plasma triglycerides (TG), (C) plasma cholesterol, (D) plasma free fatty acids (FFA). Data are mean ± SEM from n=5 animals per group.

**Supplemental Table S1: Primers used for quantitative real-time PCR analysis**

| **Gene** | **Forward primer** | **Reverse primer** |
| --- | --- | --- |
| ***Mouse*** |  |  |
| Vnn1 | TATGTCTTCCCTGAAGTGTT | CCCAGTCCTTCCCATAC |
| Vnn3 | CCACAGATCGTGCTAAGT | TCTCAAACACTCTTCCATACAG |
| F4/80  Clec7a  Tnfa  Glut4  IL-1b  Gss  Gpx1  Gclm | CTTTGGCTATGGGCTTCCAGTC  AGGTTTTTCTCAGCCTTGCCTTC  CAGACCCTCACACTCAGATCATCT  GGAAGGAAAAGGGCTATGCTG  GCAACTGTTCCTGAACTCAACT  CAAAGCAGGCCATAGACAGGG  GTGCAATCAGTTCGGACACCA  AGGAGCTTCGGGACTGTATCC | GCAAGGAGGACAGAGTTTATCGTG  GGGAGCAGTGTCTCTTACTTCC  CCTCCACTTGGTGGTTTGCTA  TGAGGAACCGTCCAAGAATGA  ATCTTTTGGGGTCCGTCAACT  AAAAGCGTGAATGGGGCATAC  CACCAGGTCGGACGTACTTG  GGGACATGGTGCATTCCAAAA |
| Ppparg | CACAATGCCATCAGGTTTGG | GCTGGTCGATATCACTGGAGATC |
| Pepck | CAGGATCGAAAGCAAGACAGT | AAGTCCTCTTCCGACATCCAG |
| G6pase | GGCCTGTAATGACGGCCATTT | CCGACTGCCAATAGGAAGGT |
| ***Rat*** |  |  |
| Vnn1 | GGGAGTTTCAGGTGTTGAG | TGAGTGTGCTATGAGGTCTG |
| Vnn3 | CAAACACCCACAACACCAG | ACCTCAACTCAGACAGCAG |
| Pparg | ATGGAGCCTAAGTTTGAGTTTGCT | GGATGTCCTCGATGGGCTTCA |
| Pepck | CCACAGGATGAGGAACCGTG | CTTCGATGGGCACTCCTTCA |
| G6pase | CATGGGCACAGCAGGTGTAT | CGACATTCAAGCACCGGAATC |
